# A Review of the Current Status and Development Trends of Compression Casting Concrete

**DOI:** 10.3390/ma19091737

**Published:** 2026-04-24

**Authors:** Xiangfeng Xu, Yang Yu, Haozhe Han, Shuo Xu, Feng Zhang

**Affiliations:** 1 College of Traffic and Civil Engineering, Shandong Jiaotong University, Jinan 250061, China; 204063@sdjtu.edu.cn; 2 State Key Laboratory of Tunnel Engineering, School of Qilu Transportation, Shandong University, Jinan 250061, Chinazhangfeng2008@sdu.edu.cn (F.Z.)

**Keywords:** compression casting concrete, mechanical properties, durability, solid waste utilization, equipment, microstructure

## Abstract

This paper presents a systematic review of compression casting concrete (CCC) based on a comprehensive literature retrieval from the Web of Science, covering publications from 2020 to 2026. CCC applies pressure on fresh concrete to expel excess internal water and air, driving the cement paste to fully penetrate the aggregate pores, which can significantly optimize the micro- and macro-properties of concrete. With environmental friendliness and resource-saving merits, CCC has become a global research hotspot in the field of civil engineering and construction. Research contributions have been made by scholars from China, Australia, Pakistan, France, the UK, India, Italy and other regions. This paper systematically elaborates the basic principles and core advantages of the compression casting technology, focusing on the analysis of key research directions, including mechanical properties, ductility improvement, durability, solid waste resource utilization (waste rubber particles, recycled concrete aggregates), compression-casting-reinforced concrete members and special-purpose preparation equipment. It analyzes the advantages and disadvantages from both micro and macro perspectives and demonstrates the engineering application feasibility and development potential of this technology. It is concluded that the mechanical properties of CCC with compressive strength exceeding 60 MPa still require further in-depth investigation, compression casting technology improves the utilization efficiency of red mud, durability research on CCC remains insufficient, and specialized equipment for large-scale reinforced concrete CCC members needs further development.

## 1. Introduction

High-performance concrete is widely used in civil engineering. Concrete compactness [1] affects not only its strength but also its durability, including freeze–thaw resistance and impermeability. Based on a review of advances in concrete material science, the compactness of concrete can be improved through the following approaches: ① Mix proportion design: Pores in concrete mainly result from the evaporation of residual water after cement hydration. Powers [2] proved that reducing the water–cement ratio can reduce concrete porosity. However, an excessively low water–cement ratio results in a stiff mixture that is difficult to construct, limiting further reduction in the water–cement ratio and improvement of compactness [3]. Water-reducing admixtures [4] improve mixture workability by dispersing cement particles, enabling a lower water–cement ratio and thus higher compactness. In addition, ultra-fine mineral admixtures promote cement hydration and densify the matrix, thereby enhancing concrete performance [5]. ② Curing process optimization: High-temperature and high-pressure curing [6] (with a steam pressure of 1.7–3 MPa and temperature of 200 °C) accelerates the secondary hydration of cementitious materials and improves concrete compactness. ③ Preparation process optimization: Vibration compaction can improve concrete strength by 20–30% [3]. Vacuum dewatering [7] is a technique that removes excess water from fresh concrete using a vacuum pump, improving compactness and strength. Roller-compacted concrete [8,9] is a technology that reduces concrete porosity and improves strength by compacting zero-slump concrete with vibratory rollers. However, roller-compacted concrete is mainly used in dam engineering. Extending such technologies to bridges, tunnels, slopes, and highways to enhance concrete strength remains challenging. Besides roller-compacted concrete, centrifugal concrete uses centrifugal force to achieve layered distribution and improved compactness, which is widely used in the production of piles, poles, piers, and other components, offering low water–cement ratio and high impermeability. China has successively developed prestressed concrete piles and large-diameter post-tensioned pipe piles [10], and the Chinese standard GB/T 19496-2004 [11], implemented in 2004, specifies the main equipment, core drilling, core sample processing, compressive strength test of core samples, calculation of estimated compressive strength of concrete in core sample specimens, and evaluation of test results for the core-drilling inspection of compressive strength of centrifugal high-strength concrete. Centrifugal concrete has been widely adopted in China due to its high production efficiency and low cost [12]. ASTM C1089 [13] is the standard specification for centrifugally cast prestressed concrete poles. This standard does not provide special correction factors or correction formulas for centrifugal concrete. In 2022, the large-diameter centrifugal concrete precast pier technology pioneered by Wu Pingping’s team was incorporated into the industry design code of the Ministry of Transport and applied in projects such as the Hong Kong–Zhuhai–Macao Bridge. The centrifugal forming process still has issues such as non-uniform component cross-sections and surface watermark defects.

Allen et al. [14] experimentally investigated the effect of compressive stress on concrete compressive strength. Their results showed that compressive pressure can significantly improve the compressive strength of concrete, especially for concrete with a high water–cement ratio. Nematzadeh [15,16] added different contents of excess water to concrete mixtures and then applied compression. Results showed that compressed concrete can effectively remove excess water, ensuring satisfactory workability without sacrificing mechanical properties. Some scholars have adopted a construction technology of applying pressure during concrete curing [17,18]. However, it is worth noting that this curing method greatly increases project costs; it is applicable to simple, small-scale members, but its suitability for large-scale reinforced concrete members remains to be investigated. Zhang et al. [19] studied recycled fine aggregates through compression casting, combined with the “secondary hydration” of insufficiently hydrated cement in the fine aggregates. It was concluded that the improvement in concrete strength caused by compression was more significant than that induced by secondary cement hydration.

Wu et al. [20] first invented CCC. CCC exerts high pressure on fresh concrete to expel air and excess water, thereby increasing the compactness of concrete and enabling it to achieve higher performance, lower cost and lower carbon emissions. It also paves the way for the use of recycled materials such as recycled aggregates and waste tire rubber particles in high-quality concrete. Compared with conventional concrete with the same mix proportion, CCC increases strength by 30–80% and durability by 30–90%. Compared with concrete of the same strength level, it reduces cement consumption by 25–50%. This technology also offers the advantage of waste recycling and reduces carbon emissions by 21–45%. Such a technological innovation has the potential to reshape the future of the concrete manufacturing industry.

The goal of this review is to systematically summarize the fundamental principles, mechanical properties, durability, solid waste utilization, equipment development, and existing challenges of compression casting concrete (CCC), so as to clarify the research progress and future development trends of this emerging high-performance concrete technology. This review is intended for researchers, structural engineers, material scientists, precast concrete manufacturers, and technical standards developers who are engaged in high-performance concrete, green building materials, and high-efficiency prefabricated construction technologies.

## 2. Literature Searching and Filtering Procedures

**Figure 1:**
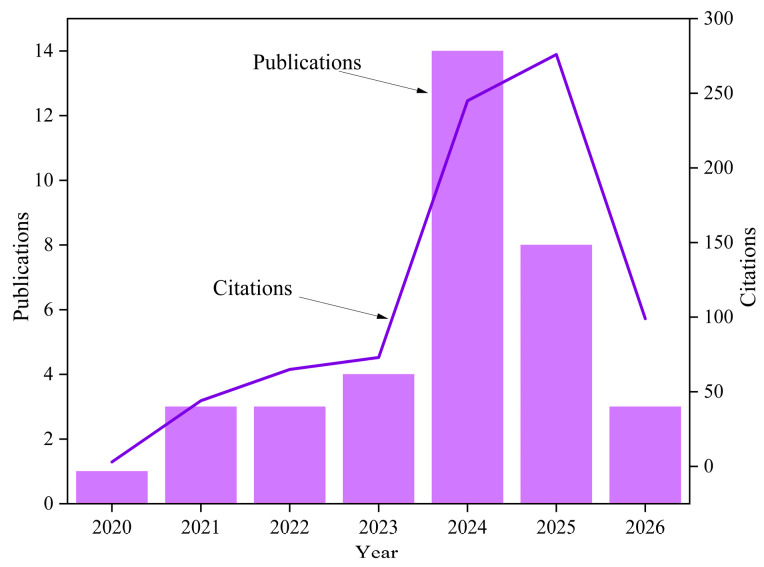
Relationship between the number of published papers, citation counts and publication year.

**Figure 2:**
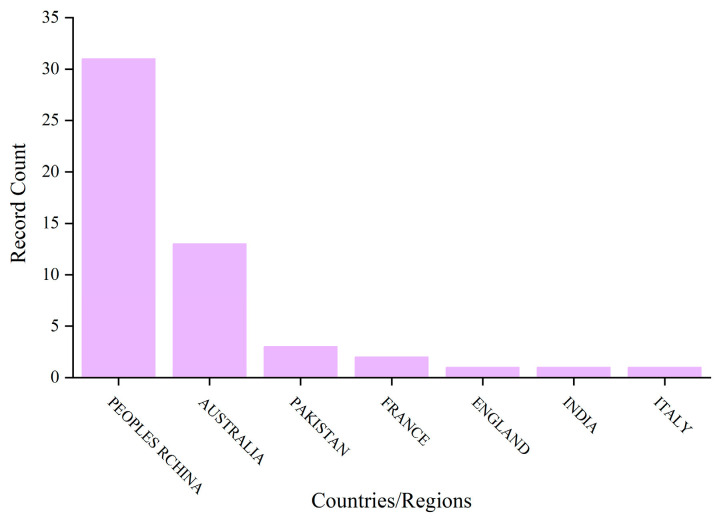
Regional distributions of the selected papers.

A rigorous literature retrieval and filtering method was adopted to ensure the validity and efficiency of information. “Web of Science” was used as the search database. The search criteria were set as follows: the term “compression casting concrete” appears in the title, and the publication date ranges from 1 January 2020 to 8 April 2026, yielding a total of 36 papers. The subject categories were restricted to “Engineering Civil”, “Construction Building Technology”, “Materials Science Multidisciplinary”, “Environmental Sciences”, and “Engineering Environmental”. After careful reading of their full contents, papers published from 2020 to 2026 and their citation counts were analyzed (Figure 1). Meanwhile, the countries corresponding to the authors’ affiliations were also analyzed (Figure 2). It can be observed from Figure 1 that the first paper on compression casting concrete was published in 2020, and the number of publications reached 14 in 2024 with a total citation count of 245. This indicates that academic studies on compression casting concrete have attracted increasing attention from researchers worldwide. As shown in Figure 2, the contributing authors are affiliated with institutions in the People’s Republic of China, Australia, Pakistan, France, England, India, and Italy.

## 3. Mechanical Properties of Compression Casting Concrete

**Figure 3:**
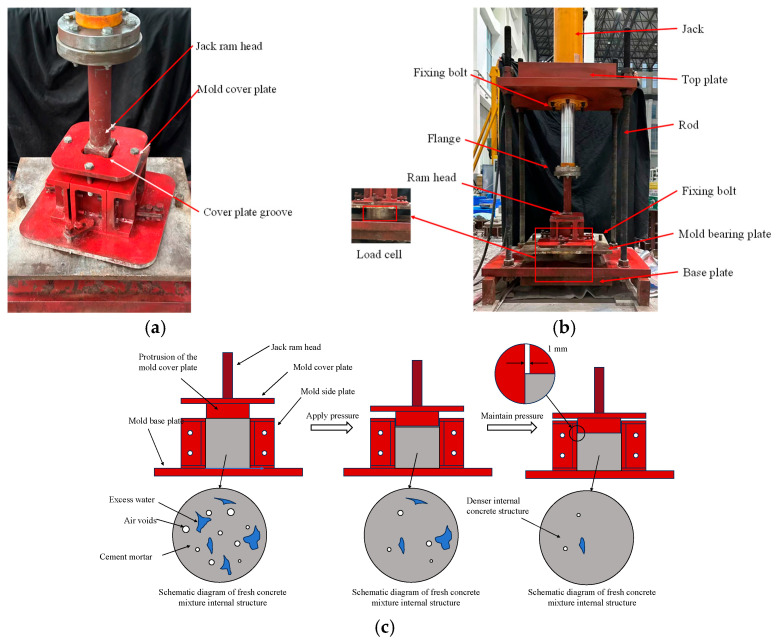
Compression casting concrete equipment and theory. (**a**) Steel mold (photo by Shuo Xu); (**b**) compression equipment (drawn by Shuo Xu); (**c**) compression principle (drawn by Shuo Xu).

Current laboratory studies on CCC are mainly focused on small-scale concrete specimens. The concrete mixture is placed into a specially designed steel mold (Figure 3a) and vibrated using a small vibrator. The mold filled with the mixture is then placed on a compression casting device for loading (Figure 3b), and pressure is stopped once the preset compression duration is reached. The purpose of compression casting is to expel air and excess water from the mixture under external pressure (Figure 3c). To ensure uniform pressure distribution, axial compression is required, which is ensured by two design measures: first, a groove is set on the top of the mold cover (Figure 3a), allowing the jack head to fit into the cover so that the jack pressure can be uniformly transferred to the mold top plate and the upper surface of the concrete; second, the inner cavity of the mold is designed to be larger than the protruding part of the cover (Figure 3c). In addition to the above mold design measures, since pressure is applied to concrete in its fresh state, the flowing concrete will naturally tend to be uniformly stressed as long as the overall device is kept horizontal.

### 3.1. Ordinary Compression Casting Concrete

**Figure 4:**
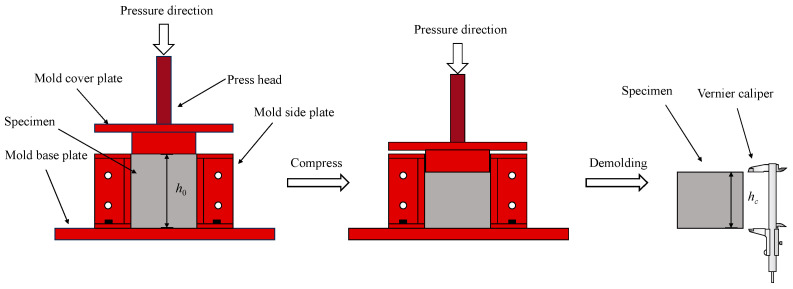
The test of the volume compression coefficient (drawn by Shuo Xu).

Xu et al. [21] carried out a study to determine the mix proportions of concrete specimens. The design was based on the volumetric mathematical model for concrete mix design [3]. In the casting process of all CCC specimens, the filling height of the concrete mixture was kept level with the height of the mold, and after curing, a vernier caliper with an accuracy of 0.1 mm (Figure 4) was used to measure the height of each CCC specimen after compression. The volume compression coefficient was calculated as shown in Equation (1):

(1) $$V_{c}=\frac{h_{0}-h_{c}}{h_{0}}$$

where $h_{0}$ is the initial height of specimen, $h_{c}$ represents the height of the specimen after compression, and $V_{c}$ represents the volume compression coefficient.

For the same concrete strength grade, CCC can significantly reduce cement consumption. Therefore, CCC exhibits a distinct advantage in reducing carbon emissions. The max cement saving ratio was 50% compared with NC [22].

Wu et al. [23] and Wang et al. [24] conducted a study on the stress–strain curves of CCC. Based on existing concrete stress–strain models, they performed regression analysis on the experimental results and obtained a stress–strain relationship model for CCC. On this basis, the equivalent rectangular stress block parameter model for flexural members of CCC was derived using the bending theorem. The research results can provide a theoretical basis for the flexural design and analysis of CCC members. Xie [25] investigated the low-speed dynamic mechanical properties of compression-cast limestone-calcined clay-based cement concrete. However, current indoor tests have all been conducted on small concrete samples, and for large CCC members, the uniformity of concrete after compression casting requires further in-depth research. Several compressive strength prediction models for CCC have been developed, and the specific models are presented in Table 1. As can be seen from Table 1, the compressive strength of the reported CCC specimens is still less than 60 MPa, and further investigation is needed to establish strength prediction models for CCC with compressive strength higher than 60 MPa in future studies. Compared with conventional concrete, the upper limit of the compressive strength enhancement ratio of CCC also varies significantly (81.25%, 122.17%, 94.12%).

Liang et al. [26] modified recycled aggregate compression cast concrete using nano-silica. Nanomaterials can not only fill interfaces, pores, and cracks but also act as nucleation sites for crystallization during cement hydration. In addition to the above beneficial effects, nano-silica particles also possess the extra function of pozzolanic reaction [27]. Therefore, nano-silica can further improve the microstructure and mechanical properties of recycled aggregate concrete (RAC), and the overall performance of nano-silica modified RAC can be increased by up to 130% [28], making nano-SiO_2_ one of the most commonly used nanoparticles for RAC modification. Kang et al. [29] investigated the nanoscale mechanism of compression casting using fully atomistic molecular dynamics simulations of the C-S-H/rubber interface [30]. Surface roughness was introduced into the adopted C-S-H model, providing a more realistic representation of the cement surface compared with existing studies. The results show that sufficient pre-compression significantly improves interfacial integrity. As the pre-compression pressure increases from 100 atm to 4000 atm, the peak pull-out force of the C-S-H/rubber interfacial transition zone increases by 90.26%, and the interfacial binding energy increases by 56.65%. Collectively, these studies at the micro and nano scales reveal the core mechanism of compression casting technology: by optimizing the interfacial transition zone (ITZ) structure, filling internal pores, and promoting cement hydration, CCC fundamentally improves the mechanical and durability performance of concrete from the meso- and micro-levels. The application of nanomodification and molecular dynamics simulation not only verifies the enhancement effect of compression casting on the microstructure of concrete but also provides a theoretical basis for the performance optimization and engineering application of CCC, especially for recycled aggregate concrete and rubber concrete with inherent interface defects.

### 3.2. Ductility Enhancement Techniques for CCC

**Figure 5:**
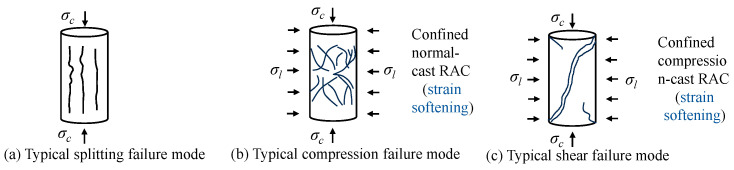
Three failure modes (drawn by Feng Zhang).

CCC can effectively improve the strength of NC, but the compressed concrete exhibits increased brittleness [26]. To improve the ductility of CCC, Yi [31] used polypropylene fibers to mitigate the brittleness of CCC. The results show that the descending branch of the compressive stress–strain curve of CCC specimens is steeper and shorter than that of uncompressed concrete, indicating brittle behavior. The addition of polypropylene fibers can significantly reduce such brittleness and enhance the post-peak compressive stress–strain behavior of CCC. Compared with uncompressed plain concrete, fiber-reinforced CCC specimens exhibit significant improvements in compressive strength, elastic modulus, and toughness (by 68%, 34%, and 38%, respectively). Yuan et al. [32] used spiral stirrups to enhance the ductility of CCC. The results show that after adopting the compression casting technique, steel spiral-confined conventionally cast RAC exhibits typical compressive failure (Figure 5), accompanied by a stress–strain curve characterized by strain hardening, whereas steel spiral-confined compression-cast RAC shows local shear failure (Figure 5), accompanied by a stress–strain curve characterized by strain softening. Stress–strain models for steel spiral-confined compression-cast RAC and conventionally cast RAC were established (Equation (2)):

(2) $$\left\{\begin{array}{l} \frac{f_{cc}}{f_{co}}=1+5.42f_{l}^{-0.11}(\frac{f_{l}}{f_{co}}{)}^{a}\\ a=f_{co}^{0.06}\\ \frac{\varepsilon_{cc}}{\varepsilon_{co}}=1+13.1(\frac{f_{l}}{f_{co}})\\ \frac{\varepsilon_{cu}}{\varepsilon_{co}}=1.27+700\varepsilon_{cu}(\frac{f_{l}}{f_{co}}) \end{array}\right.$$

where $f_{cc},f_{co},f_{l},\varepsilon_{cc},\varepsilon_{co}$ and $\varepsilon_{cu}$ represent the compressive strength of CCC confined by steel spirals, the compressive strength of unconfined RAC, the confining pressure induced by the yielding of steel spirals, the axial strain of CCC confined by steel spirals, the axial strain of unconfined RAC, and the ultimate axial strain, respectively.

**Figure 6:**
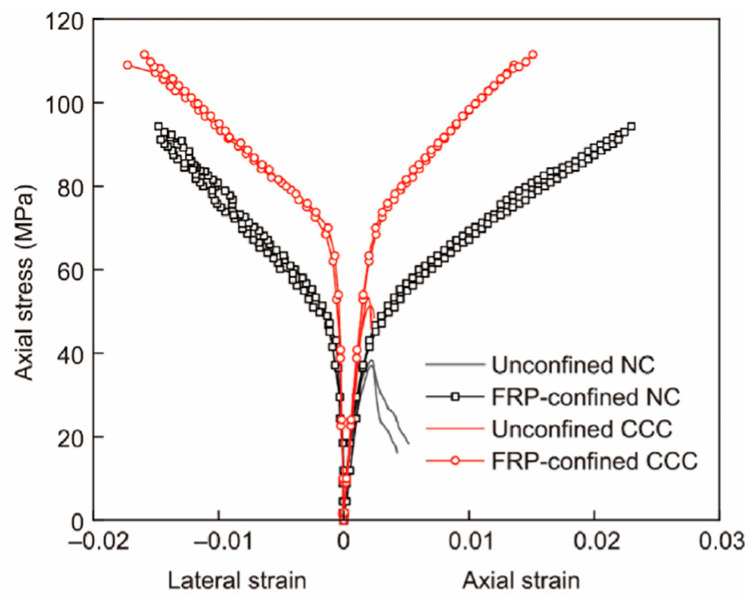
Axial and hoop strain test results of FRP-wrapped NC and CCC [33].

**Figure 7:**
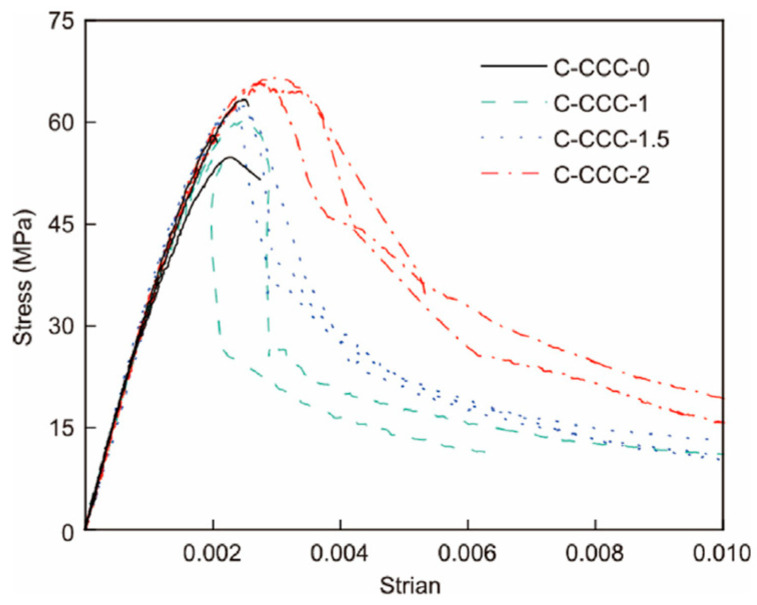
Stress–strain curves of CCC with different steel fiber volume fractions [33].

Wu et al. [33] presented test results of cylindrical specimens of NC, CCC, and corresponding fiber-reinforced polymer (FRP) confined samples. The results indicate that the ductility of both NC and CCC is significantly improved after FRP confinement (Figure 6). Due to the high brittleness of CCC, the descending branch of the stress–strain curve for CCC columns without steel fibers barely exists (specimens are crushed shortly after the peak load). As the steel fiber content increases, the descending slope gradually decreases. A larger steel fiber volume fraction (Figure 7) leads to a gentler descending branch of the stress–strain curve and better ductility. Li et al. [34] investigated the mechanical properties of compression-cast steel fiber-reinforced concrete under static and dynamic loads. The addition of steel fibers improved ductility, with the ultimate strain increased by 53% (static) and 16.8% (dynamic), respectively. Steel fiber can mitigate the brittle behavior of CCC. However, further in-depth research is still required to develop prediction models for the mechanical properties of steel fiber-reinforced CCC, including its strength, elastic modulus and peak strain.

**Figure 8:**
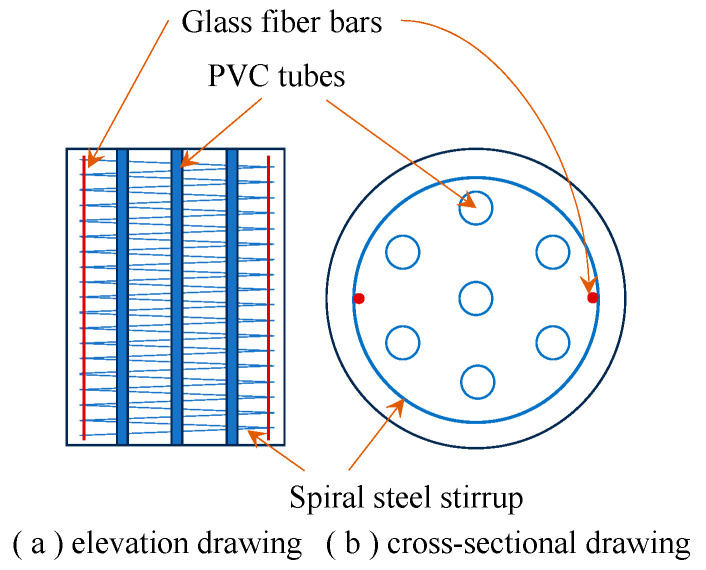
Structure of high-ductility CCC (drawn by Feng Zhang).

**Figure 9:**
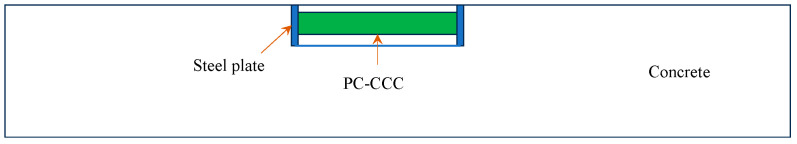
Ductile beam structure using high-ductility CCC cylinders (drawn by Feng Zhang).

Jiang et al. [35] proposed a high-ductility configuration for compression casting concrete in which steel fibers were incorporated into concrete with internal reserved holes and stirrups arranged. The detailed configuration is shown in Figure 8. A simplified trilinear stress–strain model was proposed. This device was developed to form a high-ductility structural device in the compression zone of reinforced concrete beams (Figure 9), so as to comprehensively improve the ductility of RC beams. It is worth noting that the study in Ref. [35] truly initiated the application research of CCC in reinforced concrete structural engineering. However, the applied research of CCC still needs to be further strengthened.

### 3.3. Solid Waste Application in Compression Cast Concrete

Kazmi et al. [36] explored the application of waste tire rubber and recycled aggregates [37] in CCC. Although replacing 20% natural aggregate with crumb rubber reduced the compressive strength of recycled aggregate concrete by 49%, compression casting effectively restored its strength and elastic modulus to levels comparable with conventional concrete. Yuan et al. [38] focused on FRP-confined compression-cast recycled aggregate concrete and noted that FRP confinement significantly increased the ultimate axial stress, while compression casting slightly reduced FRP fracture strain without weakening confinement efficiency. Compared with simple recycled aggregate concrete, the combined use of CCC and FRP confinement provides a more effective way to improve both strength and ductility.

Wu et al. [39] further confirmed that compression casting significantly enhanced the compressive strength, elastic modulus, and stress–strain behavior of rubberized concrete. Even with 20% crumb rubber replacement [40], the mechanical properties of CCC exceeded those of natural aggregate concrete, and rubber replacement could reach 30% while retaining satisfactory performance. Unlike conventional rubberized concrete, which usually suffers from serious strength loss, CCC enables high-volume waste rubber utilization while maintaining superior mechanical performance. All the above studies demonstrate the potential of compression-casting concrete in reducing carbon emissions [41,42].

Zheng et al. [43] and Farooq [44] provided comparative findings on compression stress. Zheng et al. [43] reported that the strength enhancement became insignificant when compression stress exceeded 5 MPa, whereas Farooq [44] observed a nearly proportional strength improvement up to 30 MPa. In Farooq’s experiment, internally saturated surface-dry recycled aggregates were used as raw materials with a water–cement ratio of 0.25. Considering that 1 g of cement requires 0.23 g of water for complete hydration, and the aggregates can still absorb part of the water in the internally saturated surface-dry state, the water–cement ratio in this paper is relatively low, which leads to the inconsistency between the strength improvement law and that in Ref. [43]. The contradictory results reveal that the optimal compression pressure is highly dependent on mixture parameters, particularly the water–cement ratio and aggregate state. Farooq [44] also conducted a comparative analysis of the compressive strength at 28 days and 7 days, and the 28-day strength was 1.57 times the 7-day strength.

Red mud [45] is an industrial waste residue generated during the extraction of alumina from bauxite. Due to its high alkalinity and large output, it poses a serious threat to the environment. Millions of tons of red mud are produced worldwide every year, and its main disposal method is stockpiling, which not only occupies a large amount of land but also may cause water and air pollution. In view of this, the use of red mud as a cement substitute in concrete has become an important direction for current research on architectural and environmental sustainable development. We carried out preliminary experimental research. The materials used in the test included cement, river sand, crushed stone, water, and red mud. The cement adopted was Conch Brand P.O 42.5 ordinary Portland cement. The river sand was medium sand and was used after drying. Crushed stone with a single particle size gradation of 5–10 mm was used and employed after washing and drying. The red mud was provided by Shandong Hi Speed Group. A total of five cylindrical specimens with a height of 200 mm and a diameter of 100 mm were cast: three specimens of red-mud-based compression cast concrete (CCC) and two specimens of normally cast concrete (NC). The concrete mix proportions are listed in Table 2. A compression stress of 5 MPa and a compression duration of 5 min were adopted for the compression cast specimens.

**Figure 10:**
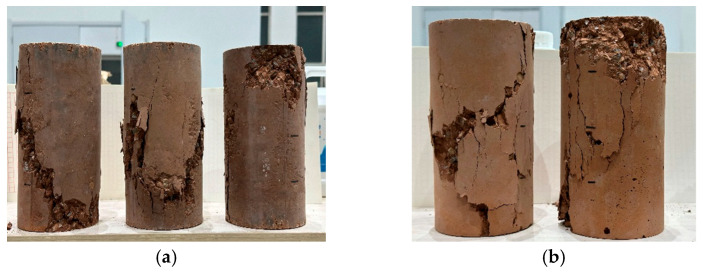
Failure modes of red mud CCC and red mud NC. (**a**) Red mud CCC; (**b**) red mud NC (photo by Shuo Xu).

The failure modes of red-mud-based CCC specimens and red-mud-based NC specimens after axial compression are shown in Figure 10. As can be seen from Figure 10, both red-mud-based CCC and red-mud-based NC specimens develop longitudinal diagonal cracks after failure; the CCC specimens have a single concentrated wide crack running through the entire specimen, while the NC specimens have scattered cracks with multiple fine cracks.

**Figure 11:**
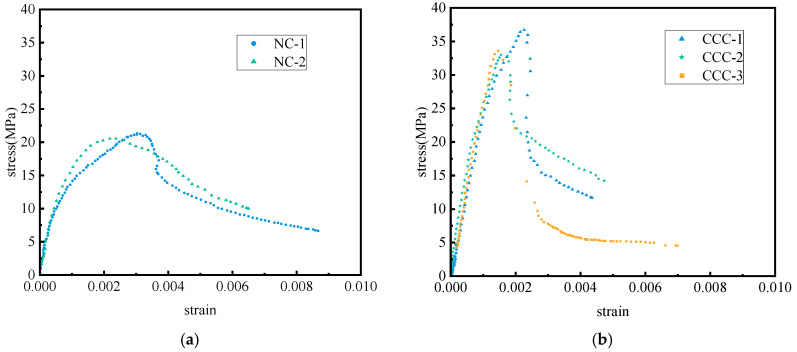
Stress–strain curves of red mud CCC and red mud NC. (**a**) Red mud CCC; (**b**) red mud NC (Drawn by Shuo Xu).

The stress–strain data of red-mud-based CCC and red-mud-based NC are shown in Figure 11. Compared with red-mud-based NC, the ascending branch of red-mud-based CCC is steeper and has a higher peak stress. In contrast to red-mud-based NC, the data points on the descending branch of red-mud-based CCC are sparse, indicating a rapid stress decrease, and the specimen fails due to the loss of rapid load-bearing capacity. However, this phenomenon is not observed in red-mud-based NC; the data points on its descending branch are relatively dense and continuous, and the specimen gradually loses its load-bearing capacity. This is because, compared with NC specimens, CCC specimens have higher compactness and lower porosity after compression casting, showing more characteristics of brittle failure. The compressive strengths of NC are 21.3 MPa and 20.6 MPa, respectively, while those of CCC are 36.8 MPa, 33.0 MPa, and 33.7 MPa, respectively. The preliminary test results show that the compressive strength of CCC is 1.64 times that of NC.

### 3.4. Compression Cast Concrete with Seawater, Sea Sand and Desert Sand

The combination of compression casting technology with seawater, sea sand, and desert sand represents an important approach to addressing the conflict between the shortage of building materials and environmental protection. River sand is essential for concrete, but its annual production consumes 50 billion tons of resources, resulting in shortages of river sand and environmental problems. Desert sand (DS), which covers 6 million square kilometers, can help alleviate this scarcity, yet its performance is relatively poor. Kazmi et al. [46] developed high-strength desert sand concrete (DSC). Nine concrete mixtures with different DS replacement ratios (0, 50, 100%) and designed strengths (30, 50, 70 MPa) were prepared. Compared with conventional concrete, compression casting increased the compressive strength and splitting tensile strength of DSC by 93% and 54%, respectively. It also reduced water absorption and porosity by up to 41% and 34%. The cost, carbon dioxide emissions, and energy consumption of compression-cast DSC were reduced by 57%, 43%, and 42%, respectively.

Reinforced concrete (RC) structures in coastal and marine regions face two major challenges [47]: shortage of raw materials and harsh service environments. Concrete made with sea sand and seawater (SS SW) can address the shortage of natural sand by using locally available resources. Meanwhile, the newly developed Compression Casting Technology (CCT) has significantly improved the micro and macro properties of concrete and enhanced its durability under harsh environments. Therefore, combining SS SW concrete with CCT to form a new type of compression cast SS SW concrete can simultaneously solve the problems of material shortage and poor durability for coastal RC structures.

Compression cast concrete fails rapidly after the peak load and exhibits greater brittleness than conventional concrete, which may affect the seismic performance of columns. The axial brittleness of seawater sea sand concrete columns [48] has been improved by using FRP confinement. Wu et al. [49] conducted an in-depth study on the micro-properties of compression-cast seawater sea sand concrete. CCT had no substantial effect on the hydration of seawater sea sand concrete. CCC was particularly effective in improving the interfacial transition zone structure for pores larger than 50 nm, enhancing the mechanical properties of seawater sea sand concrete. These findings are practically significant for the application of seawater sea sand concrete and solve problems related to the scarcity of specific natural resources, raw material shortages, and performance issues.

To address corrosion and environmental issues and achieve green and sustainable civil engineering practices, Yuan et al. [50] designed and investigated several corrosion-resistant compression cast seawater sea sand concrete (SSC) beams reinforced with carbon fiber reinforced polymer (CFRP) bars (i.e., FRP SSC beams). Their flexural behavior was studied through four-point bending tests. The beam test results indicated that the brittleness of FRP SSC beams requires special attention.

In the future, CCC can be combined with research on solid waste utilization to further expand its research scope. In accordance with engineering requirements, relevant code development and engineering applications of solid waste utilization can also be further carried out.

## 4. Durability of Compression Cast Concrete

Yi et al. [51] investigated the freeze–thaw damage of CCC. The mechanical properties and freeze–thaw damage evolution of compression-cast fiber-reinforced concrete (CCFRC) were evaluated through rapid freeze–thaw cycle tests. Statistical test results show that no significant increase in mass loss rate was observed for CCC specimens; even negative growth in mass loss rate occurred as the number of freeze–thaw cycles increased. This can be attributed to the dense internal structure of CCC. As freeze–thaw cycles proceed, microcracks gradually form and propagate inside the concrete, causing the specimens to absorb water continuously until saturation. Consequently, the mass gained by water absorption exceeds the mass of surface mortar spalled by freeze–thaw cycles [52]. A quadratic parabola model for freeze–thaw damage deterioration of compression casting concrete (CCC) is obtained. After 150 freeze–thaw (F-T) cycles, applying a higher compaction pressure of 15 MPa during compression casting can densify CCC and improve its microstructural characteristics, thereby significantly enhancing its mechanical properties and durability. The freeze–thaw damage of CCFRC is less severe than that of CCC.

**Figure 12:**
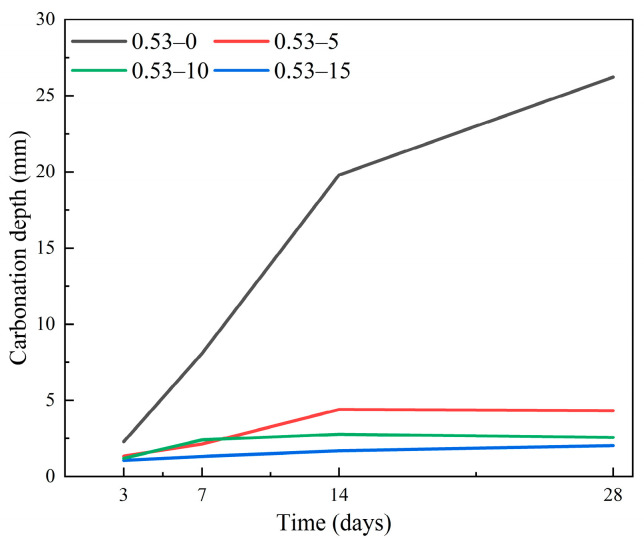
Effect of compressive stress on carbonation depth [53].

Li et al. [53] measured the carbonation depth of all CCC specimens and uncompressed concrete specimens by spraying phenolphthalein solution on the split surfaces after CO_2_ exposure. The carbonation depth of CCC specimens was smaller than that of uncompressed specimens, and the improvement in carbonation resistance was consistent with the compressive strength results (Figure 12). Therefore, CCC can be adopted in reinforced concrete structures to minimize the penetration of carbon dioxide into concrete.

Xiong [54] investigated the mechanical properties of CCC under coupled sulfate attack and dry–wet cycles. Within 60 days of erosion, the mass and relative dynamic elastic modulus of CCC showed a slight increase, while compressive strength decreased slowly. Higher water–cement ratio and sulfate concentration accelerated the erosion process by increasing salt crystallization and erosion products. Compared with uncompressed normal concrete (NC), CCC exhibited smaller mass change (indicating better impermeability) and consistently higher compressive strength before and after erosion, though no clear short-term trend was observed for its relative dynamic elastic modulus.

Compared with studies on the mechanical properties of CCC, durability findings on CCC are relatively limited. There is still great potential for durability research on CCC. Further in-depth studies are needed on the performance of CCC under freeze–thaw cycles [55], chloride ion erosion [56], carbonation, fire resistance, impact resistance and abrasion resistance.

## 5. Equipment and Technology

In existing experimental studies, all cast specimens are small-scale components, and compression casting can be readily achieved using simple equipment consisting of a jack, reaction frame, and pressure sensor. However, applying such equipment to large-scale structural members (e.g., beams and columns) is difficult. These components have large compression areas and large compression depths, which not only require higher formwork strength and stiffness but also significantly increase production costs. To address this issue, researchers have proposed corresponding solutions [50,57].

**Figure 13:**
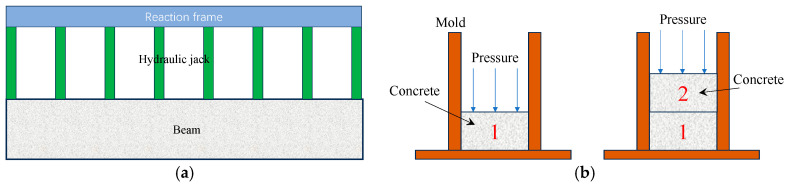
Synchronous compression casting and layered compression casting. (**a**) Simultaneous compression casting; (**b**) layered compression casting (drawn by Feng Zhang).

(1) To address the problem of a large compression area, a multi-jack synchronous compression casting technique can be adopted, as illustrated in Figure 13a. For beam-type members, the use of a single jack not only demands a high load capacity but also tends to cause uneven compression of the beam due to insufficient stiffness of the top formwork during compression casting. In contrast, the multi-jack synchronous compression casting method arranges multiple jacks uniformly along the longitudinal direction of the beam and controls them to perform compression casting simultaneously via a program. This approach not only reduces the required load capacity of individual jacks but also ensures that the beam is uniformly compressed along its length.
(2) Wang et al. [57] proposed a layered compression casting (LCC) technique (Figure 13b) and provided recommended LCC parameters, including compression stress, casting layer height, and compression duration.

**Figure 14:**
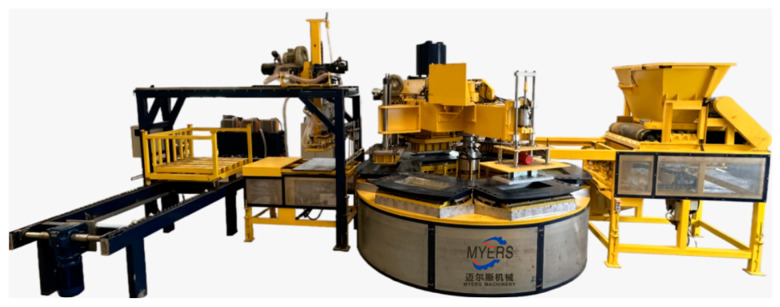
Six-station compression casting equipment for stone-like components.

Technical research and development based on the Changshu Myers equipment (Figure 14), and the iterative innovation of compression casting equipment, can provide key support for the large-scale application of large size compression casting concrete components.

**Figure 15:**
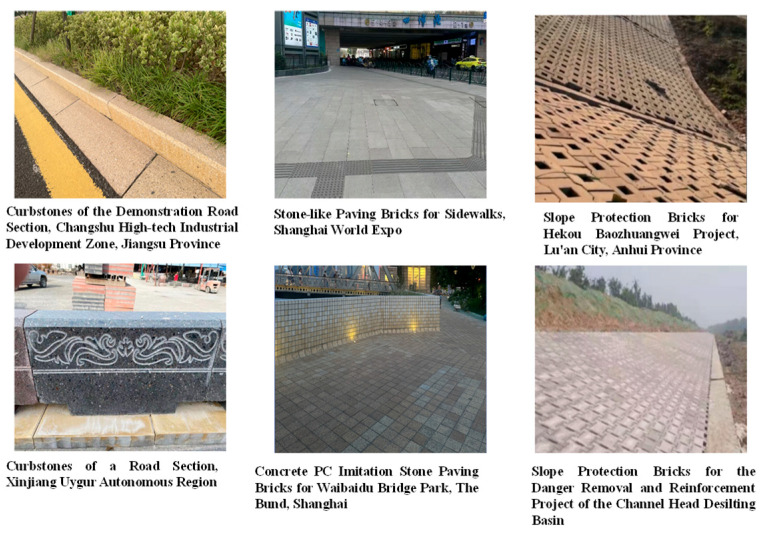
Engineering application of stone-like concrete components prepared by wet pressure filtration molding (image provided by Changshu Myers Machinery Co., Ltd., Changshu, China).

It is worth noting that similar equipment for compression casting of large size concrete components already exists in the engineering field. Myers Company has carried out R&D on compression casting engineering equipment for curbstones, stone bricks, slope protection bricks, and other products (Figure 15).

**Figure 16:**
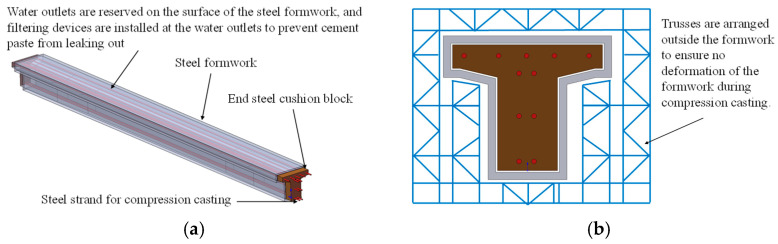
Construction Scheme for casting of 13 m short T-beams via compression method. (**a**) Schematic diagram of the compression process; (**b**) steel truss for external formwork reinforcement (drawn by Shuo Xu).

**Figure 17:**
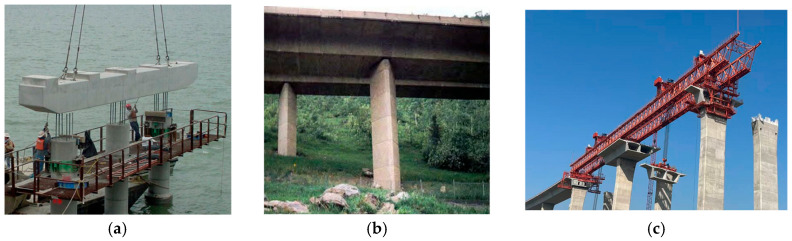
Precast reinforced concrete member for bridges. (**a**) Prefabricated piers of Lake Ray Hubbard Bridge [59]; (**b**) Vail Pass Bridge, Colorado, USA [60]; (**c**) prefabricated segmental box girders of Zhangjinggao Bridge (photo by Shuo Xu).

For longer-span beams, the authors suggest that the compression casting technology can be further developed in combination with prestressing tension technology. In this paper, a preliminary compression casting scheme is proposed for the 13 m span low-profile T-beam commonly used in bridge engineering (Figure 16), and the compression casting of the 13 m low-profile T-beam is explained as follows with reference to the standard drawings of low-profile T-beams [58]. The side, top and bottom formworks of the 13 m low-profile T-beam are made of steel formwork, and custom steel bearing plates with the same size as the end cross-section of the T-beam are adopted at the ends. To avoid lateral deformation of the formwork during compression casting, the outer side of the formwork is reinforced with trusses. To ensure the discharge of water and air during compression casting, water outlets are uniformly arranged on the surface of the steel formwork, and filtering devices are installed at the water outlets to prevent cement paste from seeping out. The compression force for compression casting is applied by tensioning the steel strands arranged inside the beam. Based on the above description of the compression casting technology using prestressed steel strands, the compression casting process can be further promoted to the manufacturing of engineering components such as segmental prefabricated bridge piers and box girders (Figure 17).

In recent years, with the mechanization and rapid construction development of construction technology, construction requirements have been continuously raised. To effectively shorten the construction period of building projects, researchers have focused on the rapid curing of concrete members. Steam curing [61] has become the most commonly used curing method because steam [62] is characterized by high heat content, high humidity, convenient production and transportation, and low cost and can effectively promote the strength development of concrete when properly used. It has been gradually accepted in large-scale engineering applications, especially in projects with restricted construction cycles, where preliminary applications and explorations have been carried out. Existing bridge engineering cases [63] show that high-temperature steam curing for 24 h can make the strength of T-beams for bridges reach the 7-day design strength. As an important technology for precast concrete members, compression casting concrete technology needs to be further studied in combination with high-temperature steam curing technology in the future.

## 6. Advantages and Disadvantages of Compression Cast Concrete

Compression-cast concrete (CCC) improves the macro and micro properties of concrete through a physical compaction process applied to fresh mixture, instead of chemical additives or material modification. Based on the above review of mechanical properties, durability, structural behavior, and equipment development, this section systematically summarizes the main advantages and existing limitations of CCC technology to provide a comprehensive reference for its engineering application and future research.

### 6.1. Advantages

CCC exhibits prominent advantages in mechanical performance, durability, sustainability, and production efficiency. Due to the elimination of excess water and air under external pressure, the internal microstructure becomes denser, and the interfacial transition zone between aggregate and cement paste is significantly improved. Consequently, CCC presents higher compressive strength, elastic modulus, and load-bearing efficiency than conventional concrete with the same mix proportions. Meanwhile, the compact internal structure effectively inhibits the penetration of corrosive media, leading to superior durability including enhanced carbonation resistance, freeze–thaw stability, chloride penetration resistance, and water tightness.

From an environmental and economic perspective, CCC allows a remarkable reduction in cement dosage while satisfying the required strength, which directly lowers material costs and reduces carbon emissions generated during cement production. Another outstanding merit is its strong compatibility with recycled or inferior materials, such as recycled aggregates, waste rubber, recycled brick/glass powder, desert sand, and red mud. Even at high replacement ratios, CCC can restore or even exceed the performance of natural aggregate concrete, thereby promoting large-scale resource utilization of solid wastes. In addition, owing to the short pressure-holding duration and fast demolding speed, CCC supports high-efficiency prefabrication and improves mold turnover rate in industrial production.

### 6.2. Disadvantages and Technical Challenges

Despite the above superiorities, CCC still faces several limitations that restrict its widespread application. The most notable issue is the increased material brittleness. The highly densified microstructure leads to a steeper descending branch in the stress–strain curve and lower ultimate compressive strain, which reduces the ductility and deformability of structural members.

Furthermore, the implementation of compression casting relies on specialized loading devices and high-stiffness formworks to resist forming pressure, which increases the technical difficulty and production cost, especially for large-scale members. Uniform pressure distribution over large components remains difficult to guarantee, potentially leading to inconsistent material performance. In addition, CCC is more suitable for precast components rather than cast-in situ construction, which limits its application scenarios.

At present, design codes and standards for CCC are still lacking, and conventional concrete design methods cannot fully reflect its brittle characteristics and stress–strain behavior. Most existing studies are conducted under laboratory conditions, and long-term performance data under real service environments, such as long-term loading, fatigue, and complex environmental actions, remain insufficient.

### 6.3. Summary

In summary, compression-cast concrete provides a novel, high-performance, low-carbon, and sustainable technical route for the concrete industry. Its comprehensive advantages in mechanical properties, durability, resource utilization, and environmental benefits are remarkable. However, issues including material brittleness, equipment dependence, forming technology for large members, lack of design specifications, and insufficient long-term performance data must be systematically addressed through further research. With continuous improvement in preparation technology, design theory, and construction equipment, CCC is expected to be widely applied in prefabricated structural engineering and green construction.

## 7. Conclusions

From the extensive studies on CCC materials, the main conclusions of this study are as follows:

(1) In 2020, Professor Wu Yufei invented the CCC technology. By applying high pressure to fresh concrete to expel air and excess water, this technology effectively improves the compactness of concrete, thereby endowing it with superior performance, lower cost, and lower carbon emissions. Such a technological innovation is expected to reshape the development pattern of the concrete manufacturing industry to a certain extent.
(2) At present, many scholars have carried out relevant studies on the mechanical performance and design models of CCC. However, existing laboratory tests are all based on small-scale concrete specimens. For large-scale CCC members, the uniformity of concrete after compression casting still needs to be further investigated.
(3) The combination of CCC and fibers can improve the toughness of concrete to a certain extent. Configuring stirrups in the compression zone of reinforced CCC members can effectively enhance their ductility. This research has truly opened the chapter of applied research on CCC in reinforced concrete structural engineering. Nevertheless, studies on the engineering application of CCC to reinforced concrete (RC) beams still need to be further strengthened.
(4) Current research on the durability of CCC mainly focuses on sulfate attack. Studies on chloride attack, carbonation, fire resistance, freeze–thaw cycles, impact resistance, and abrasion resistance are still insufficient and require systematic investigation. Therefore, there remains considerable research space in the field of CCC material durability experiments.
(5) Research on CCC combined with solid waste utilization can expand the research scope of CCC technology. This study presents the first investigation on compression cast red mud concrete. The experimental results show that compression casting technology can effectively improve the material utilization value of red mud.
(6) In existing experimental studies, all cast specimens are small-scale members, and compression casting can be realized using simple equipment composed of jacks, reaction frames, and pressure sensors. However, it is rather difficult to apply similar simple equipment to large structural members commonly used in engineering, such as beams and columns. Such members feature a large compression area and great compression depth, which not only impose higher requirements on the strength and stiffness of CCC formwork but also lead to a significant increase in production costs. Therefore, the research and development of compression casting equipment will become one of the core technical challenges for the engineering application of CCC technology.

## Figures and Tables

**Table 1:** CCC Compressive Strength Model.

Source	CCC Compressive Strength Prediction Model	Considered Factors	CCC Compressive Cylindrical Strength (MPa)	Strength Improvement Ratio (%)
Wang et al. [24]	$f_{ccc}=0.121\rho_{ccc}-252.15$	Concrete density *ρ_ccc_* (kg/m^3^)	29–52	26.83–81.25
Wang et al. [24]	$f_{ccc}=-49.092w/c+70.478$	Water–cement ratio *w*/*c*	29–52	26.83–81.25
Wang et al. [24]	$f_{ccc}=-0.0374f_{nc}^{2}+3.0058f_{nc}-9.7349$	Strength of ordinary concrete *f_nc_* (MPa)	29–52	26.83–81.25
Wu et al. [22]	$\begin{array}{l} f_{\mathrm{CCC},\mathrm{IV}}=(-59.228w/c+36.610V_{c}+44.159)\\ \times (27.888{\left(w/c\right)}^{2}-17.067w/c+4.664) \end{array}$	Water–cement ratio *w*/*c*; the volume compression coefficient *V_c_*	28.84–57.48	25.97–122.17
Wu et al. [23]	$f_{ccc}=-0.014C^{2}+0.9814C-120.05$	Cement content *C* (kg/m^3^)	29–52	26.83–94.12

**Table 2:** Mix proportions.

Material	Cement (kg/m^3^)	Sand (kg/m^3^)	Coarse Aggregate (kg/m^3^)	Water (kg/m^3^)	Red Mud (kg/m^3^)
Dosage	433	545	970	221	241

## Data Availability

The original contributions presented in this study are included in the article. Further inquiries can be directed to the corresponding author.
